# Electro-oxidation of formoterol fumarate on the surface of novel poly(thiazole yellow-G) layered multi-walled carbon nanotube paste electrode

**DOI:** 10.1038/s41598-021-92099-x

**Published:** 2021-06-17

**Authors:** N. Hareesha, J. G. Manjunatha

**Affiliations:** Department of Chemistry, FMKMC College, Constituent College of Mangalore University, Madikeri, Karnataka India

**Keywords:** Chemistry, Analytical chemistry, Electrochemistry

## Abstract

The current study explicates the electro-oxidation behavior of formoterol fumarate (FLFT) in the presence of uric acid (UA) on the surface of poly thiazole yellow-G (TY-G) layered multi-walled carbon nanotube paste electrode (MWCNTPE). The modified (Poly(TY-G)LMWCNTPE) and unmodified (MWCNTPE) electrode materials were characterized through electrochemical impedance spectroscopy (EIS), field emission scanning electron microscopy (FE-SEM), and cyclic voltammetry (CV) approaches. The characterization data confirms the good conducting and electrocatalytic nature with more electrochemical active sites on the Poly(TY-G)LMWCNTPE than MWCNTPE towards the FLFT analysis in the presence of UA. Poly(TY-G)LMWCNTPE easily separates the two drugs (FLFT and UA) even though they both have nearer oxidation peak potential. The electro-catalytic activity of the developed electrode is fast and clear for FLFT electro-oxidation in 0.2 M phosphate buffer (PB) of pH 6.5. The Poly(TY-G)LMWCNTPE offered a well-resolved peak with the highest electro-oxidation peak current at the peak potential of 0.538 V than MWCNTPE. The potential scan rate and oxidation peak growth time studies show the electrode reaction towards FLFT electro-oxidation is continued through a diffusion-controlled step. The variation of concentration of FLFT in the range from 0.2 to 1.5 µM (absence of UA) and 3.0 to 8.0 μM (presence of UA) provides a good linear relationship with increased peak current and a lower limit of detection (LOD) values of 0.0128 µM and 0.0129 µM, respectively. The prepared electrode gives a fine recovery for the detection of FLFT in the medicinal sample with acceptable repeatability, stability, and reproducibility.

## Introduction

Among today’s generation, a considerable number of people are suffering from lungs related health issues, such as asthma, lung cancer, chronic obstructive pulmonary disease, influenza-related infections, respiratory failure, severe acute respiratory syndrome, pneumonia, tuberculosis, and middle east respiratory syndrome^1,2^.

Asthma is the most common incurable and long-lasting inflammatory infection in the routes of the lungs, which causes erratic breathing problems to humans of all ages. Asthmatic patients suffer from irritable breathing difficulties, due to puffy and inflamed airways, chest tightness, shortness of breathing, heavy coughing, and reversible airflow obstruction^3^. Over 339 million people are suffering from asthma and more than 80% of deaths are occurring due to asthma-related problems in low and lower-middle-income countries. Even though it is not curable but giving an amalgamation of inhaled corticosteroids can control asthma by preventing the loss of lung functions. The clearing and relaxing of the muscles of airways of lungs take place to improve breathing by intake of long-acting beta-adrenoceptor agonists medications specifically like salmeterol and FLFT. Also, the COVID-19 epidemic is so scary for asthmatic patients, it is essential to know that presently there is no confirmation of increased infection rates in asthma patients. Only one article reveals that asthma can upsurge the hospitalization from COVID-19 in adults (this data is based on a small number of patients)^4^. While considering the data from New York it reveals that asthma was defending the persons who died from COVID-19^5^. FLFT is also termed as (E)-but-2-enedioic acid; N-[2-hydroxy-5-[(1R)-1-hydroxy-2-[[(2R)-1-(4-methoxyphenyl) propan-2-yl] amino] ethyl] phenyl] formamide, is a salty fumarate of formoterol. FLFT is a long-acting and sympathomimetic β2-receptor agonist applied in the inhaler activity during the treatment, control, or prevention of some lung diseases like allergic airway disease, chronic obstructive pulmonary disease, and asthma^6–8^. FLFT connects β2-receptor agonist in respiratory smooth muscle and accelerates the intracellular adenylyl cyclase with the development of 3′,5′-cyclic adenosine monophosphate. This produced component initiates the relaxation of smooth muscles in the lungs. Nonetheless, in certain circumstances, FLFT reveals some side-effects such as dizziness, headache, nausea, anxiety, increased dehydration or urination, stomach upset, weariness, irregular heartbeat, weakening of the muscle, rising of blood pressure, and some severe allergic reactions in the face, tongue, and throat. Also, FLFT overdose causes some serious difficulties in the living body such as trouble while breathing, chest pain, irregular heartbeat, nervousness, and muscle cramps in the living organisms. This data highlights the necessity of a sensitive and stable FLFT analyzing tool in commercial and pharmaceutical samples.

Until now, a few analytical approaches such as gas chromatography^9^, liquid chromatography-mass spectrometry^10^, thin-layer chromatography, high-performance liquid chromatography (HPLC)^11^, ion-pair chromatography (IPC)^12^, spectrophotometry^13^, reversed-phase high performance liquid chromatography (RP-HPLC)^14^, and capillary electrophoresis^15^ have been reported in the literature for the detection of FLFT in commercial and pharmacological samples. Nonetheless, these approaches have some drawbacks such as costly instrumentation, lengthy and time-consuming experimental procedures, and need pre-treatment, solvent extraction, and complex instrumentation. Hence, the analysis of FLFT significantly needs a simpler, fast, low-cost, highly sensitive, reliable, and accurate method. In comparison with these methods, electrochemical techniques are clear-cut, powerful, economical, instant, highly sensitive, selective, and consistent^16–21^.

A material used for the fabrication of working electrodes is more essential for the quantification of electroactive molecule^22–24^ such as FLFT. Until now, only a few electrochemical works were reported in the literature for the detection of FLFT at different sensing materials such as yolk–shell structured copper oxide at silica oxide spheres^25^, suberic acid-functionalized CuO nanoflowers^26^, and glassy carbon electrode^27^. These electrodes need high price, laborious pre-treatment processes, and provide a lower or nearer sensitivity and selectivity. Hence, electro-analysis of FLFT essentially requires a simple and commercial electrochemical sensor with high accuracy, consistency, sensitivity, and selectivity.

Conductive polymers (CPs) are synthesized from an easy and cost-effective electro-polymerization process, which have analogous electrical and optical characteristics as semiconductors and metals. CPs are the most widely used category of polymers for various applications in the field of diodes, batteries, electrochemical sensors, biomedical, drug delivery, and actuators. Besides, CPs reveal some distinct properties such as low-cost, fast response, redox actions, superior electron affinity, corrosion protection, lower energy optical transition and ionization potential, high ionic and electronic conductivity, easy interaction with bio-molecule, and electro-active compounds. These properties make it appropriate as a remarkable sensing material in the electro-analytical field^28^. Multi-walled carbon nanotubes (MWCNTs) have greater attention in the electro-analytical field due to their excellent electrical conductivity, catalytic action, more surface sites, high mechanical strength, high chemical and thermal stability, and electronic properties^29^. Additionally, MWCNTPE specifically has a hydrophobic surface and offers electronic and hydrophobic interactions with electro-polymerized components^30^.

Currently, a composite form of MWCNTs and CPs initiates attention in the field of electrodes and their applications in the detection of electroactive molecules. Also, MWCNTs and CP composite materials have easy functionalized nature with enhanced electrical and mechanical features, due to the contact of MWCNTs with CPs by π–π covalent, electronic, and hydrophobic interactions^31^.

In this effort, a new and conductive Poly(TY-G)LMWCNTPE was fabricated for the sensitive and selective electro-analysis of FLFT in the presence and absence of UA. The surface properties of the Poly(TY-G)LMWCNTPE and MWCNTPE materials were interpreted by FE-SEM, EIS, and CV methodologies. The electrochemical activities were achieved through differential pulse voltammetry (DPV) and CV methods. Moreover, the presented electrode is eco-friendly and has not been used in the previous reports for FLFT analysis.

## Experimental

### Apparatus and experimental measurements

Electrochemical assessments were documented using CV, DPV, and EIS methods at the electrochemical analyzer (CHI-6038E, USA) connecting with a routine tri-electrode system. A platinum electrode (Pt-wire) is the auxiliary electrode, a saturated calomel electrode (Hg_2_Cl_2_) is the reference electrode, and Poly(TY-G)LMWCNTPE and MWCNTPE are the working electrodes. CV was premeditated with the potential scan rate of 0.1 V/s, sample interval of 0.001 V, quiet time of 2.0 s, and sensitivity of 0.1 mA/V. DPV was accomplished with the amplitude of 0.1 V, sample width of 0.02 s, pulse width of 0.06 s, pulse period of 0.5 s, quiet time of 2.0 s, and sensitivity of 0.1 mA/V. Different pHs of PB were prepared using the EQ-610 instrument. Distilled water (DW) was prepared using the VITSIL-VBSD/VBDD water purifying instrument. FE-SEM pictures of modified and unmodified electrodes were achieved using the ZEISS instrument (DST-PURSE Lab, Mangalore University, India).

### Chemicals

FLFT (≥ 98%) was purchased from Sigma-Aldrich, India. UA (99%), TY-G (colorant ≥ 98%), MWCNTs (exterior diameter of 30–50 nm and length of 10–30 μm), and silicone oil (binder, 98%) were taken from Molychem, India. Na_2_HPO_4_·2H_2_O (99.5%), NaH_2_PO_4_·H_2_O (99%) and K_4_[Fe(CN)_6_]·3H_2_O (98.5%) were bought from Himedia, India. KCl (99.5%) was procured from Nice Chemicals, India. All the used chemicals were of analytical reagent grade and used with no additional refinement. All the required solutions of known concentration were prepared by suspending them in a known amount of DW.

### Preparation of FLFT real sample solution

FLFT capsules were procured from a local pharmaceutical store. The powder present in the capsules was taken out and dissolved (with constant stirring for 30 min) in a known amount of DW. Further, the dilution of the FLFT containing sample solution was carried out using PB.

### Fabrication of MWCNTPE and Poly(TY-G)LMWCNTPE

The MWCNTPE was fabricated by blending 60% of MWCNTs with 40% of silicone oil for around 20 min to get a fine paste. The developed paste was sorted into the vacant outlet (3.0 mm diameter) of a Teflon tube and smoothly rubbed on tissue paper to get a smooth surface.

The Poly(TY-G)LMWCNTPE was fabricated through the electro-polymerization process. Here, the electro-polymerization of 1.0 mM TY-G in PB (0.2 M and 7.0 pH) was done on the external surface of MWCNTPE by cycling twenty CV segments having the potential range of − 0.60 to 1.40 V at the potential scan rate of 0.1 V/s. Later, the prepared electrode surface was rinsed carefully in DW to eliminate the unreacted monomer content.

### Electrochemical setup

Electrochemical measurements were accomplished for 0.01 mM FLFT in 0.2 M PB. The working electrode (MWCNTPE and Poly(TY-G)LMWCNTPE) immersion length was set to 1.0 cm. Each voltammetric cycle was recorded in the potential gap from 0.2 to 0.9 V against Hg_2_Cl_2_ at the potential scan rate of 0.1 V/s. This analysis was performed at 25 ± 2 °C.

## Results and discussion

### Electrode material characterization

#### FE-SEM study

**Figure 1:**
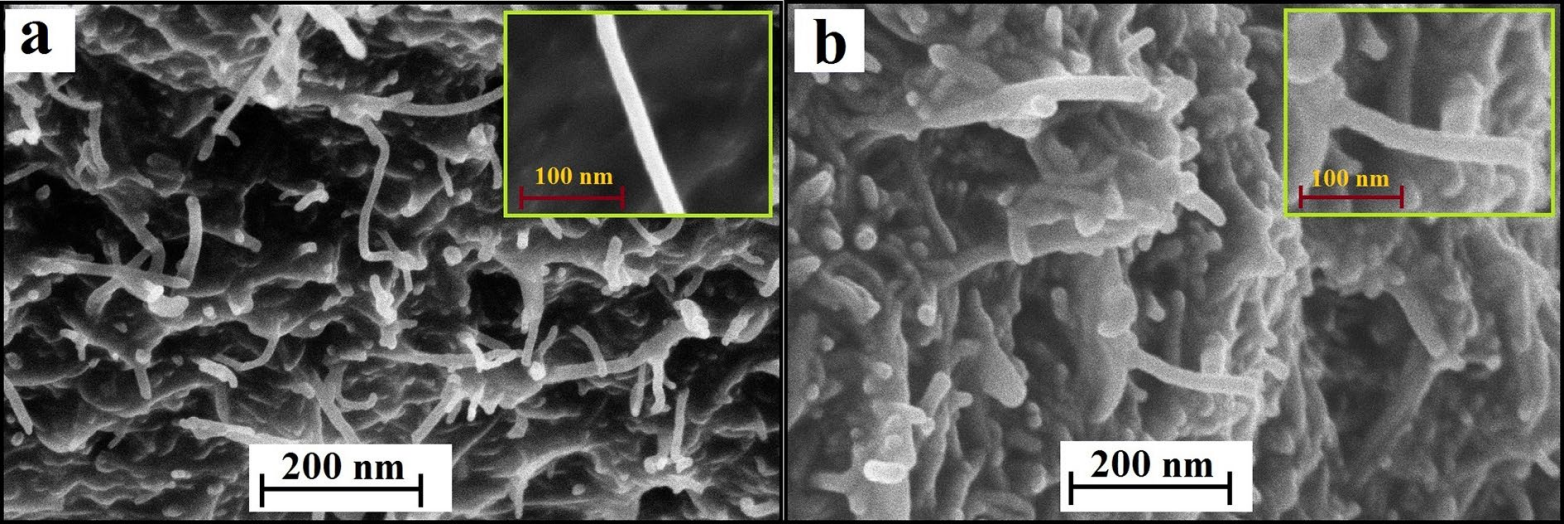
FE-SEM pictures of (**a**) MWCNTPE and (**b**) Poly(TY-G)LMWCNTPE.

The electrode surface morphological study was carried out through the FE-SEM technique. FE-SEM pictures show the surface morphology of MWCNTPE and Poly(TY-G)LMWCNTPE. Figure 1a picturizes the arbitrarily fused thin and long root-shaped tubes with erratically distributed gaps on the exterior of the electrode. Also, those tubes and gaps didn’t show any of the roofed films on their exterior (picture having 100 nm magnification), represents the surface of unmodified MWCNTPE. Furthermore, Fig. 1b displays the FE-SEM morphology of modified Poly(TY-G)LMWCNTPE. Here, each MWCNTs and erratically distributed gaps are roofed by thin deposition of an electro-polymerized film of TY-G. Moreover, the width of Poly(TY-G)LMWCNTPE is improved with sufficient porosity after the electro-polymerization of TY-G associate with MWCNT alone.

#### EIS study

**Figure 2:**
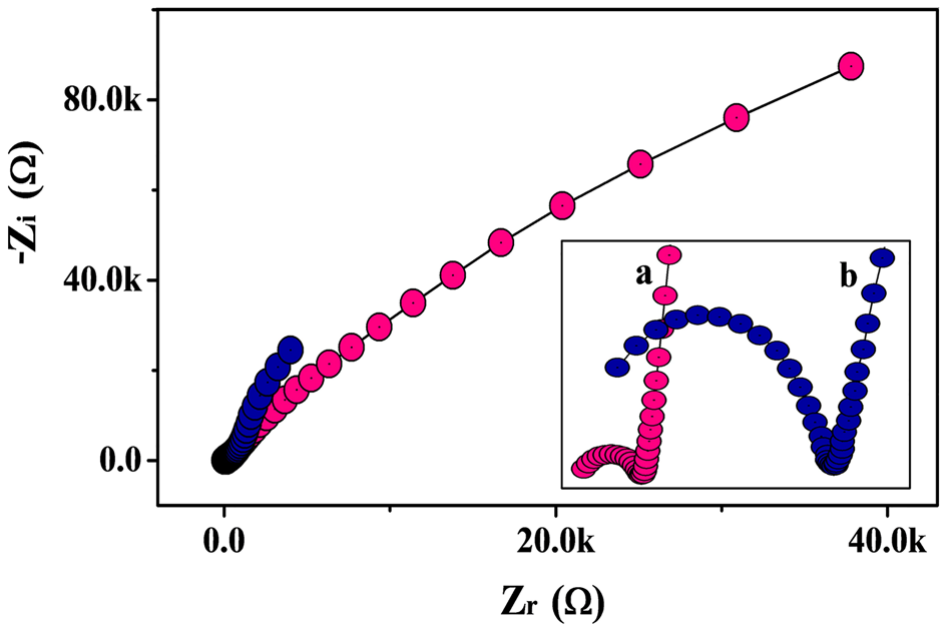
The Nyquist plots for MWCNTPE (Line ‘b’) and Poly(TY-G)LMWCNTPE (Line ‘a’).

EIS is the significant tool for the measurement of the interfacial charge transmission character of the electrode material components at the interface of the supporting electrolyte. The achieved results of EIS corresponding to MWCNTPE (Line ‘b’) and Poly(TY-G)LMWCNTPE (Line ‘a’) are presented using Nyquist plots (Inset Fig. 2). EIS experimentation was conducted in the supporting electrolyte KCl (0.1 M) having K_4_[Fe(CN)_6_]·3H_2_O (1.0 mM) at 0.06 V of working potential and 0.005 V of amplitude in the frequency range of 1.0 Hz to 100 kHz. As from the Nyquist plots, MWCNTPE affords a larger semicircle as compared to Poly(TY-G)LMWCNTPE, which specifies that the electron transfer nature of the MWCNTs is substantially enhanced by the deposition of electro-polymerized TY-G.

#### Electro-active surface area of MWCNTPE and Poly(TY-G)LMWCNTPE

**Figure 3:**
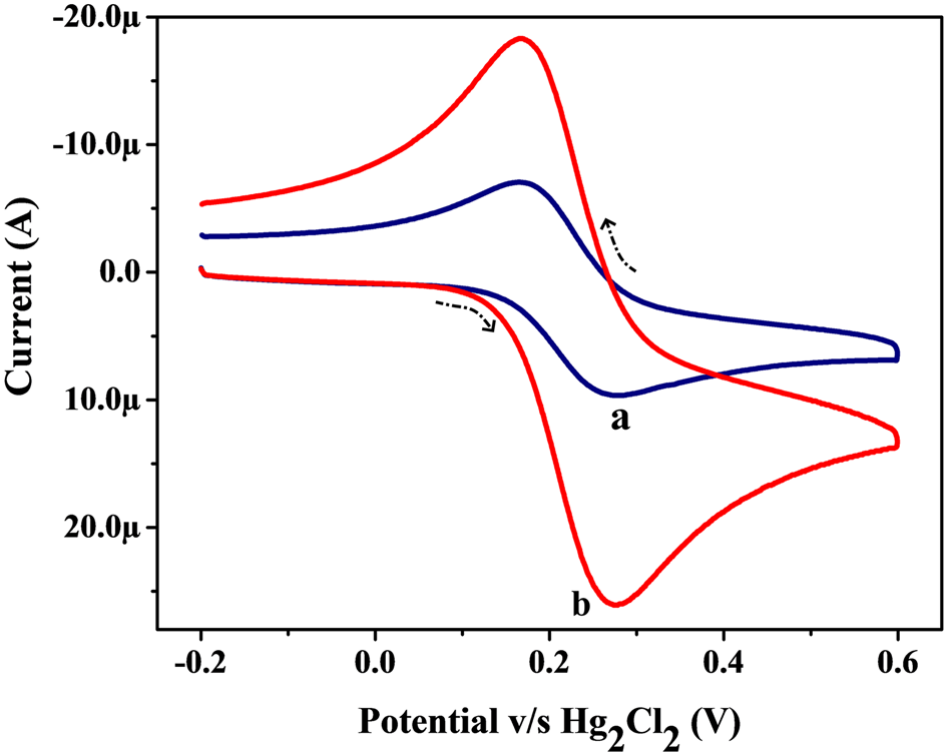
CVs for K_4_[Fe(CN)_6_]·3H_2_O (1.0 mM) in the supporting electrolyte KCl (0.1 M) on the surface of MWCNTPE (Line ‘a’) and Poly(TY-G)LMWCNTPE (Line ‘b’) at the potential scan rate of 0.1 V/s and the potential width of − 0.2 to 0.6 V versus Hg_2_Cl_2_.

Firstly, the investigative K_4_[Fe(CN)_6_]·3H_2_O solution (1.0 mM) in supporting electrolyte KCl (0.1 M) was employed with the CV method at both modified and unmodified electrodes separately. Figure 3 shows the cyclic voltammograms (CVs, at the potential scan rate of 0.1 V/s and potential width of − 0.2 to 0.6 V) for K_4_[Fe(CN)_6_]·3H_2_O at the surface of MWCNTPE (Line ‘a’) and Poly(TY-G)LMWCNTPE (Line ‘b’) in optimum conditions. It can be displayed that, the Poly(TY-G)LMWCNTPE reveals a greater redox peak current in association with unmodified MWCNTPE. The electro-active area of MWCNTPE and Poly(TY-G)LMWCNTPE surfaces was computed on the application of following Randles–Sevcik’s equation^29,32^,

1 $${\text{A}} = \frac{{{\text{I}}_{{\text{p}}} }}{{2.69 \times 10^{5} {\text{n}}^{{3/2}} {\text{~D}}^{{1/2}} \upupsilon ^{{1/2}} {\text{C}}_{0} }},$$

where, ‘A’ is the electro-active area of the electrode surface (cm^2^), ‘I_p_’ is the peak current (A), ‘n’ is the number of electrons involved in the redox reaction of K_4_[Fe(CN)_6_]·3H_2_O, ‘D’ is the diffusion coefficient (cm^2^/s) of K_4_[Fe(CN)_6_]·3H_2_O, ‘C_0_’ is the concentration (mol/cm^3^) of K_4_[Fe(CN)_6_]·3H_2_O and ‘ʋ’ is the fixed potential scan rate of CVs (V/s). The calculated electro-active surface areas of the MWCNTPE and Poly(TY-G)LMWCNTPE are obtained to be 0.0149 cm^2^ and 0.0389 cm^2^, respectively.

### Electro-polymerization of TY-G on MWCNTPE

**Figure 4:**
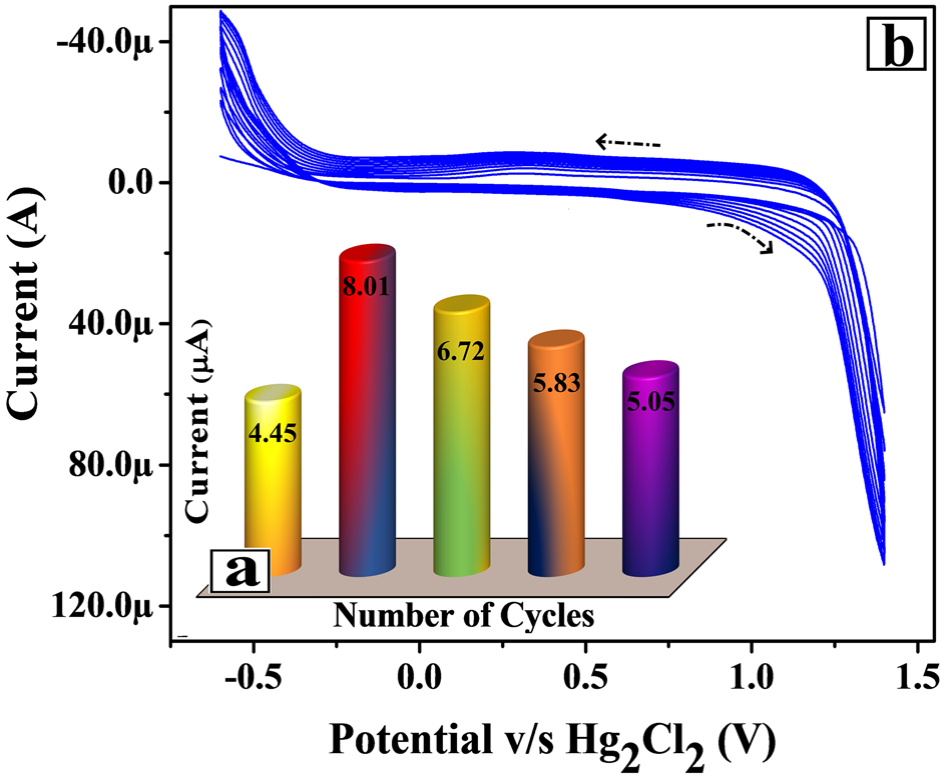
(**a**) Plot of the electro-oxidation peak current of FLFT versus the number of electro-polymerization cycles. (**b**) CVs for the electro-polymerization of TY-G (1.0 mM) in PB (0.2 M and 7.0 pH) on the surface of MWCNTPE at the potential scan rate of 0.1 V/s and the potential interface of − 0.60 to 1.40 V versus Hg_2_Cl_2_.

The polymer film thickness has a significant impact on the electrochemical feature of the Poly(TY-G)LMWCNTPE. The poly(TY-G) film thickness can be inhibited efficiently through the variation of a number of electro-polymerization cycles. Hence, the optimization of number of polymerization cycles were premeditated on the electrochemical action of FLFT (0.01 mM) in PB (0.2 M and 7.0 pH). Figure 4a displays the plot of oxidation peak current versus the number of electro-polymerization cycles. Here we observed the amplified oxidation peak current of FLFT from 5 to 10 cycles, on the other hand from 10 to 25 cycles the FLFT oxidation peak current gradually decreased, which is most feasibly due to the sufficient coverage of poly(TY-G) film on the accessible surface area of the MWCNTPE. Here, ten polymerization cycles show sufficient coverage on the surface of MWCNTPE and provide a faster rate of electron transfer with optimum peak current and the reduction of background current. As a result, ten polymerization cycles were chosen as optimum cycles for this experimentation.

**Scheme 1:**
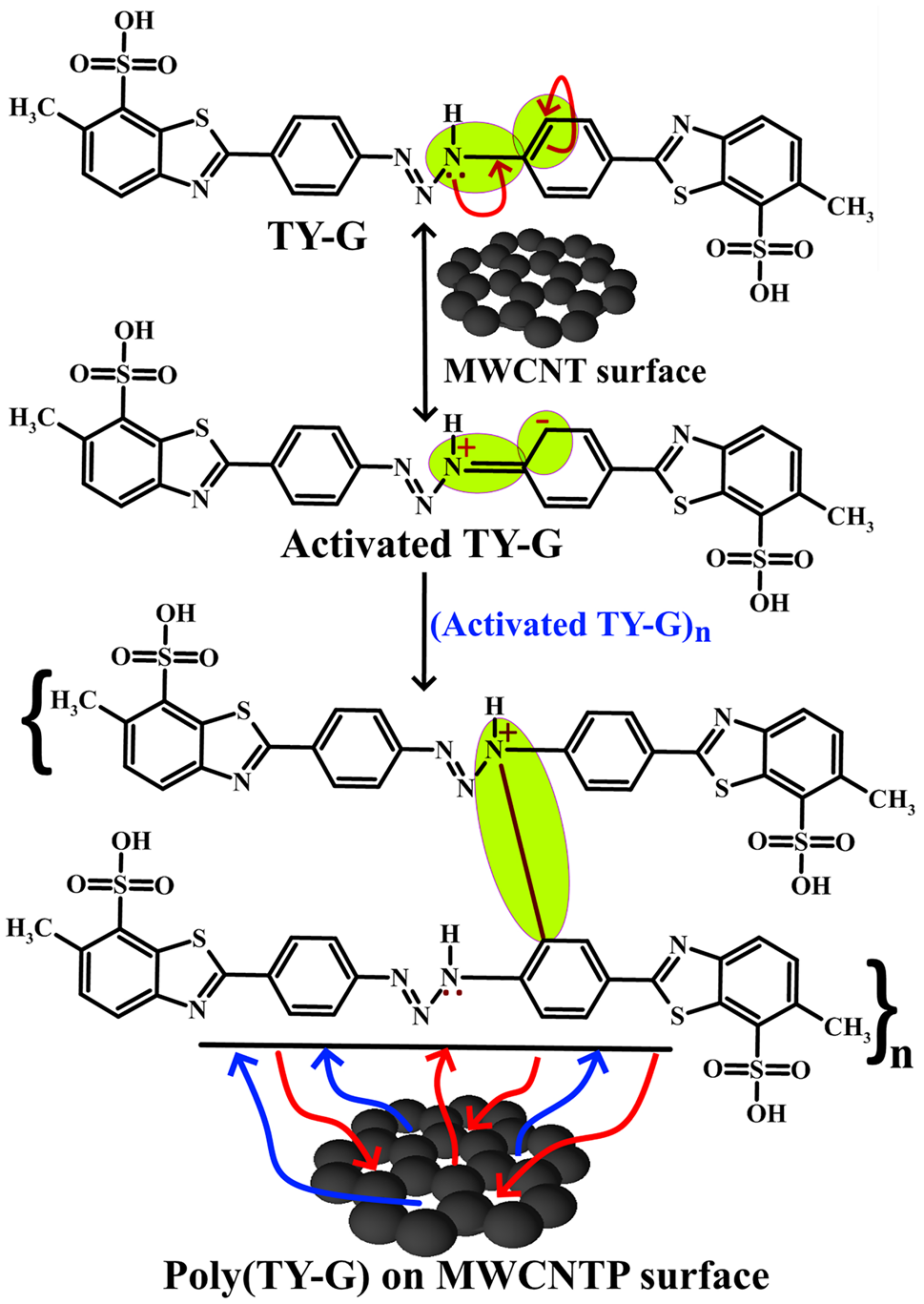
The probable reaction mechanism of electro-polymerization of TY-G on the surface of MWCNTPE.

The unification of TY-G dye polymer film on the MWCNTPE surface was done by treating 1.0 mM monomer TY-G dye solution in 0.2 M PB (supportive electrolyte) of 7.0 pH in an electrochemical chamber through the CV approach. The conversion of a monomer TY-G into a polymer of TY-G was accomplished by the electro-polymerization process. The electro-polymerization of TY-G on the MWCNTPE surface was completed using optimized ten polymerization CV cycles (at the potential interface of − 0.60 to 1.40 V and 0.1 V/s of potential scan rate) shown in Fig. 4b. Here Fig. 4b exemplifies the progressively inclined CVs during the increased cyclic time, confirms the conversion of a monomer TY-G film into an electro-polymer TY-G film. The obtained poly(TY-G) may have more π–π covalent interactions with the aromatic rings in their polymeric form and provides strong incorporation on the surface of MWCNTPE through hydrophobic interactions^33^. The probable reaction mechanism of electro-polymerization of TY-G on the surface of MWCNTPE is shown in Scheme 1.

### Impact of electrolyte pH on FLFT peak potential and current

**Figure 5:**
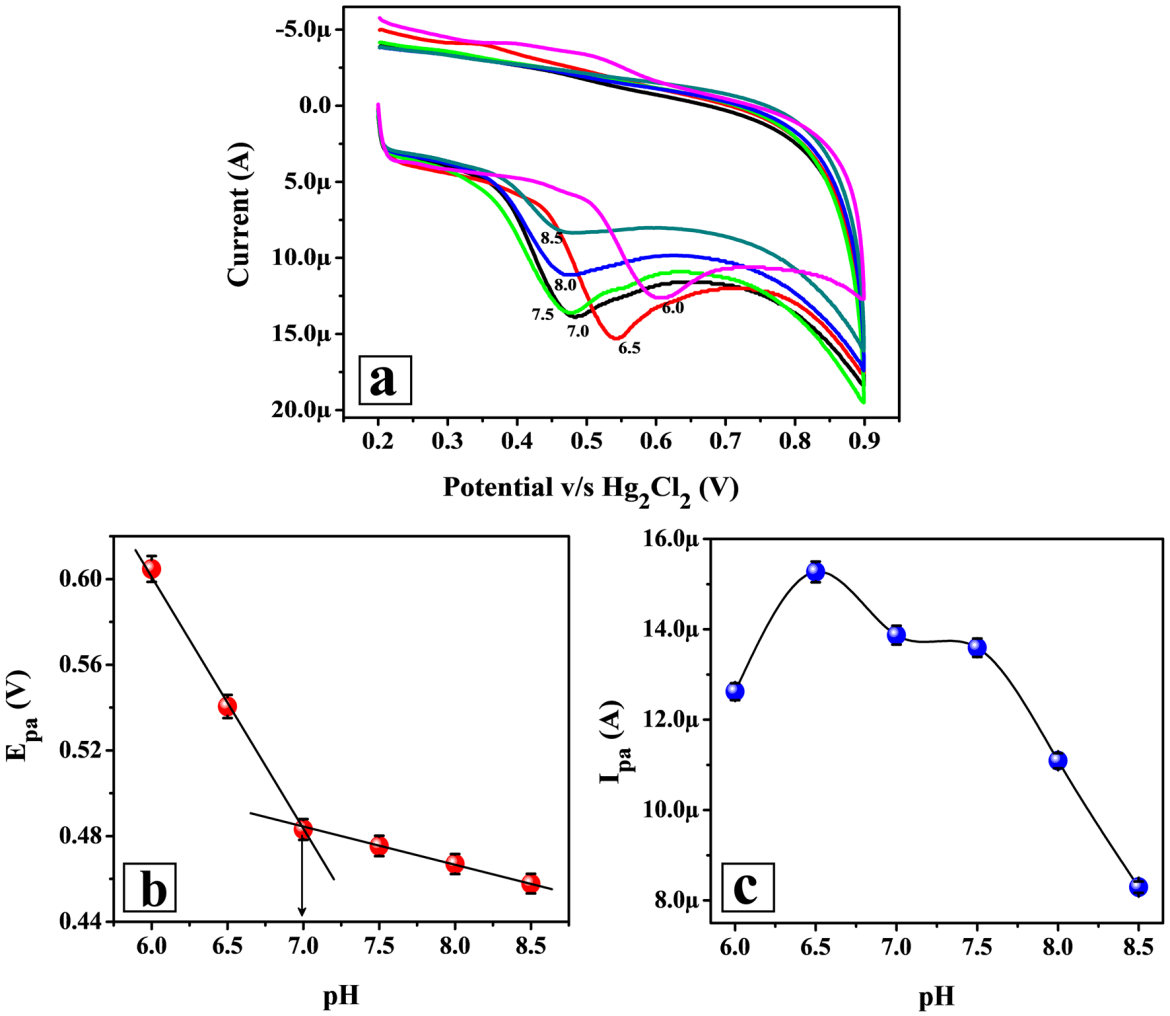
(**a**) CVs for 0.01 mM FLFT in different pHs of 0.2 M PB (6.0 to 8.5) on the surface of Poly(TY-G)LMWCNTPE at the potential scan rate of 0.1 V/s and the potential interface of − 0.60 to 1.40 V versus Hg_2_Cl_2_. (**b**) The plot of the pH of 0.2 M PB versus the electro-oxidation peak potential of FLFT (n = 6). (**c**) The plot of the pH of 0.2 M PB versus the electro-oxidation peak current of FLFT (n = 6).

The impact of supporting electrolyte pH was analyzed using the CV method (at 0.1 V/s potential scan rate and 0.2 to 0.9 V potential gap) for 0.01 mM FLFT in different pHs of 0.2 M PB (6.0 to 8.5) on the surface of Poly(TY-G)LMWCNTPE (Fig. 5a). FLFT has both amino and phenolic groups in its chemical structure which delivers the pKa values of 9.2 and 7.9 at 25 ± 2 °C, respectively. Thus, the supporting electrolyte (0.2 M PB) pH modifies the state of FLFT protonation on the surface of Poly(TY-G)LMWCNTPE. Figure 5b espied that the electro-oxidation peak potential of FLFT was transferred towards a shorter potential zone (negative shift) as the intensification of PB pH from 6.0 to 8.5. This incident specified that on the surface of Poly(TY-G)LMWCNTPE the electro-oxidation process of FLFT was a protonated electron transfer. Hence, the smaller number of protons participation supports the process of electro-oxidation with less current sensitivity at elevated PB pH (6.5 to 8.5). Fine linearity is observed between peak potential and PB pH values (plot of E_pa_ v/s pH) and the fitted linear regression equation is as follows,

2 $${\text{E}}_{{{\text{pa}}}} \left( {\text{V}} \right) = 0.903 - 0.055\,\left( { \pm \,0.001} \right)\left( {\frac{{\text{V}}}{{{\text{pH}}}}} \right)\left( {{\text{R}}^{2} = 0.903} \right),$$

where, ‘R^2^’ is the regression coefficient. Here, the slope value 0.055 V/pH is most nearer to the theoretic value (0.059 V/pH), signifying that the electro-oxidation of FLFT associates with an alike number of protons and electrons on the surface of Poly(TY-G)LMWCNTPE. Supporting this, the number of protons that participated in the electro-oxidation reaction of FLFT was determined using the following Nernst relation,

3 $${\text{B}} = \frac{{ - 2.3025\,{\text{m R T}}}}{{{\text{n F}}}},$$

where, ‘B’ is the slope of Eq. (2), ‘m’ is the number of protons that participated in the FLFT oxidation reaction, ‘n’ is the number of electrons participating in the FLFT oxidation reaction and other terms (R, T and F) represents their standard physical values. The calculated value of the number of protons was found to be 1.86 ≈ 2.

Furthermore, the pKa value of FLFT after the electro-oxidation was detected from the intersection point displayed in the plot of E_pa_ v/s pH (Fig. 5b). FLFT displays the pKa value of 7.0, indicating that the oxidation process most probably has taken place at the phenolic group of FLFT. From Fig. 5c, the electro-oxidation peak current of FLFT was very high at acidic pH of 6.5. These data are resolute that acidic pH (6.5) is most appropriate for the highly sensitive sensing of FLFT due to the strong interaction between Poly(TY-G)LMWCNTPE surface and FLFT. Therefore, the pH value of 6.5 was chosen as the optimal pH for this investigation.

### Scan rate impact on peak potential and current of FLFT at Poly(TY-G)LMWCNTPE

**Figure 6:**
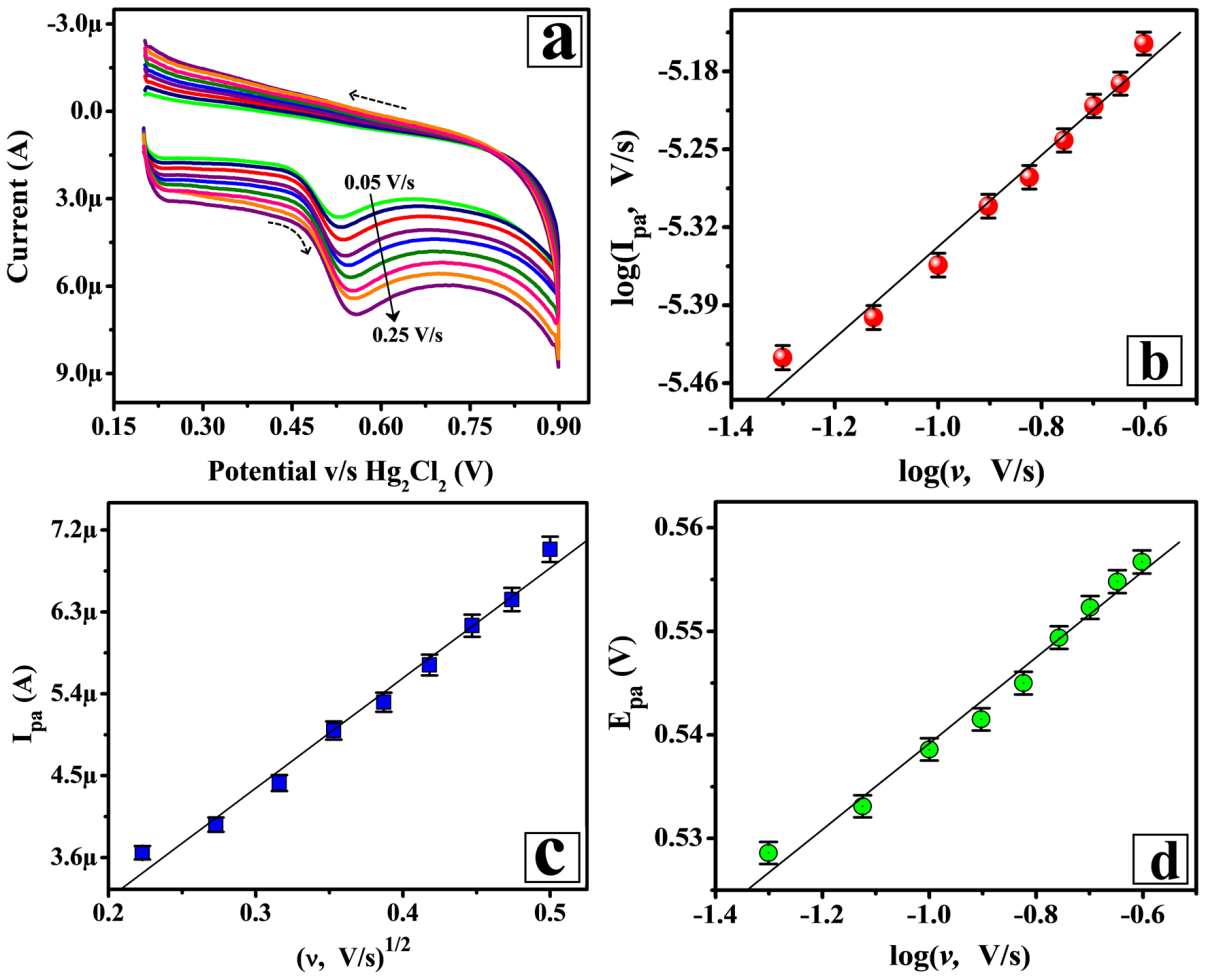
(**a**) CVs for 0.005 mM FLFT in PB (0.2 M and 6.5 pH) on the surface of Poly(TY-G)LMWCNTPE at variable potential scan rate in the range between 0.05 to 0.25 V/s and the potential window of 0.2 to 0.9 V versus Hg_2_Cl_2_. (**b**) The plot of the log value of electro-oxidation peak current versus the log value of potential scan rate (n = 9). (**c**) The plot of the electro-oxidation peak current versus the square root value of potential scan rate (n = 9). (**d**) The plot of the electro-oxidation peak potential versus the log value of potential scan rate (n = 9).

The potential scan rate impact on the peak potential and current of FLFT at Poly(TY-G)LMWCNTPE deliver a key evidence for the electro-oxidation mechanism and the kinetic behaviors of the equipped electrode. The CV executions were documented and shown in Fig. 6a for 0.005 mM FLFT in PB (0.2 M and 6.5 pH) on Poly(TY-G)LMWCNTPE at variable potential scan rate in the range between 0.05 and 0.25 V/s, which displays the increased electro-oxidation peak currents and potentials of FLFT. The kinetic nature and dependability of the modified electrode towards the oxidation reaction of FLFT were verified based on acceptable linear plots of the log value of the electro-oxidation peak current versus log value of the potential scan rate (Fig. 6b) and electro-oxidation peak current versus square root value of the potential scan rate (Fig. 6c). The obtained results are fitted in the following linear relations are as follows,

4 $${\text{log}}({\text{I}}_{{{\text{pa}}}} ,\,{\text{A}}) = 1.783 \times 10^{{ - 6}} + \left( {0.412\, \pm\, 0.023} \right)\log (\upupsilon, {\text{V}}/{\text{s}})\,\left( {{\text{R}}^{2} = 0.988} \right),$$

5 $${\text{I}}_{{{\text{pa}}}} \left( {\text{A}} \right) = 7.306 \times 10^{{ - 7}} + \left( {1.210 \times 10^{{ - 5}} \pm 5.219 \times 10^{{ - 7}} } \right) \left( {{\upupsilon, {{\text{V}}}/{{\text{s}}}}} \right)^{{{1}/{2}}} \left( {{\text{R}}^{2} = 0.993} \right).$$

The slope (0.412 ± 0.023) of Eq. (4) and the R^2^ value of Eq. (5) are very adjacent to the required theoretical values suggesting that the kinetic behavior of the electro-oxidation of FLFT on Poly(TY-G)LMWCNTPE has proceeded through a diffusion-controlled pathway^26^. The plot of electro-oxidation peak potential versus log value of the potential scan rate (Fig. 6d) provides fine linearity and the results are fitted in the following linear relation is as follows,

6 $${\text{E}}_{{{\text{pa}}}} \,\left( {\text{V}} \right) = 0.580 + \left( {0.045 \pm 0.002} \right)\log (\upupsilon, {\text{V}}/{\text{s}})\,\left( {{\text{R}}^{2} = 0.991} \right).$$

**Scheme 2:**
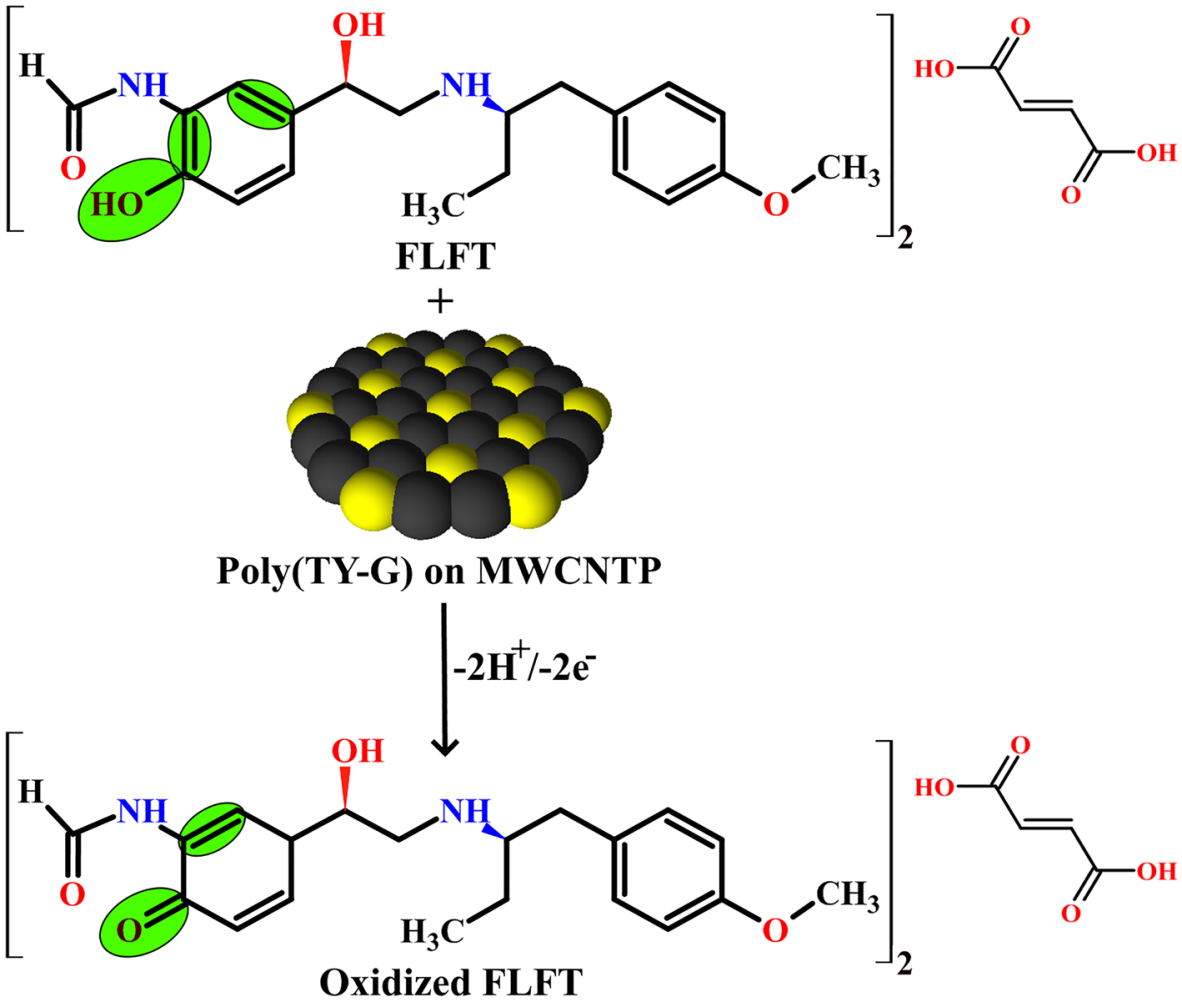
The probable electro-oxidation reaction of FLFT on the surface of Poly(TY-G)LMWCNTPE.

The number of electrons that participated in the electro-oxidation reaction of FLFT was confirmed using the slope value (0.045 ± 0.002) of Eq. (6) and the following Laviron’s and Bard-Faulkner’s relations^34,35^,

7 $${\text{E}}_{{{\text{pa}}}} = {\text{E}}^{0} + \left( {\frac{{2.3\,{\text{R T}}}}{{\left( {1 - \upalpha } \right)\,{\text{n F}}}}} \right) \log \left( {\frac{{\left( {1 - \upalpha } \right)\,{\text{n F}}}}{{{\text{R T k}}^{0} }}} \right) + \left( {\frac{{2.3\,{\text{R T}}}}{{\left( {1 - \upalpha } \right)\,{\text{n F}}}}} \right)\,{\text{log}} \upupsilon,$$

8 $${\text{E}}_{{{\text{pa}}}} - {\text{E}}_{{\frac{{{\text{pa}}}}{2}}} = \frac{{47.7}}{{\upalpha {\text{n}}}},$$

where, ‘E_pa/2_’ is the electro-oxidation peak potential at the half peak current of FLFT, ‘α’ is the electrochemical charge transfer coefficient, ‘n’ is the number of involved electrons, ‘υ’ is the applied potential scan rate, ‘k^0’^ is the electrochemical heterogeneous rate constant, and other terms (R, T, and F) represents their standard physical values. The premeditated value of a number of electrons was found to be 2.26 ≈ 2. The possible electro-oxidation reaction of FLFT is shown in Scheme 2. The electrochemical heterogeneous rate constant for the oxidation of FLFT was calculated by deducing Eq. (7) and the premeditated value of ‘k^0^’ was found to be 1.818 s^−1^.

The electrochemical surface coverage concentration (Г) of FLFT at the surface of MWCNTPE and Poly(TY-G)LMWCNTPE is calculated using the following relation^36^,

9 $$\Gamma = \frac{{\text{Q}}}{{{\text{nFA}}}},$$

where, ‘Q’ is the integrated electrical charge of the oxidation peak. The calculated value of electrochemical surface coverage concentration of FLFT for the MWCNTPE was 1.286 ÅM/cm^2^ and for the Poly(TY-G)LMWCNTPE was 2.802 ÅM/cm^2^. This consequence is the other key factor for the high electro-oxidation peak current of FLFT at Poly(TY-G)LMWCNTPE.

### Oxidation peak growth time

**Figure 7:**
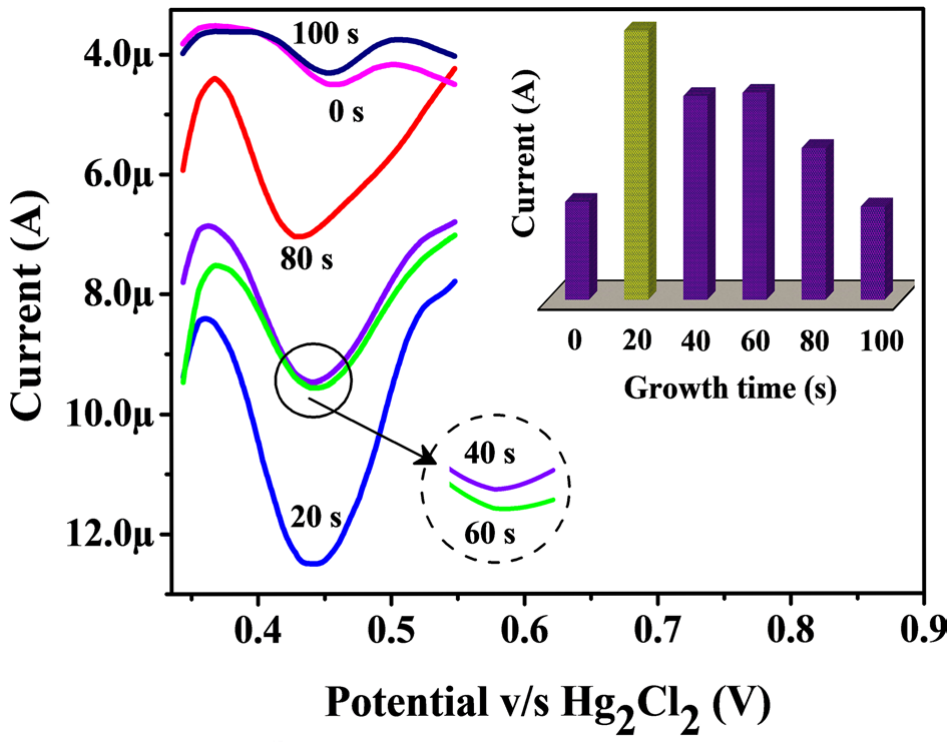
DPVs of the altered growth time (0 to 100 s) for 0.005 M FLFT in PB (0.2 M and 7.0 pH) on the surface of Poly(TY-G)LMWCNTPE and the plot of growth time versus electro-oxidation peak current of FLFT.

The influence of peak growth time is the prominent tool to disclose the maximum accumulation point (higher peak current) of FLFT on the surface of Poly(TY-G)LMWCNTPE with its kinetic behavior at the variable growth time. The peak growth time has been optimized using the DPV experimentation by altering the growth time within the range from 0 to 100 s for 0.005 mM FLFT in PB (0.2 M and 6.5 pH) and the outcomes are presented in Fig. 7. Here the observation suggests that the growth time of 20 s (exists at the second position) provides the maximum peak current than other growth times (0 s, 40 s, 60 s, 80 s, and 100 s). This consequence is most probably due to the effect of saturation followed by diffusion kinetic behavior of the equipped Poly(TY-G)LMWCNTPE rather than adsorption kinetics. Hence, twenty seconds were chosen as an optimum growth time for the FLFT analysis throughout the experiment.

### Electro-oxidation of FLFT at Poly(TY-G)LMWCNTPE and MWCNTPE

**Figure 8:**
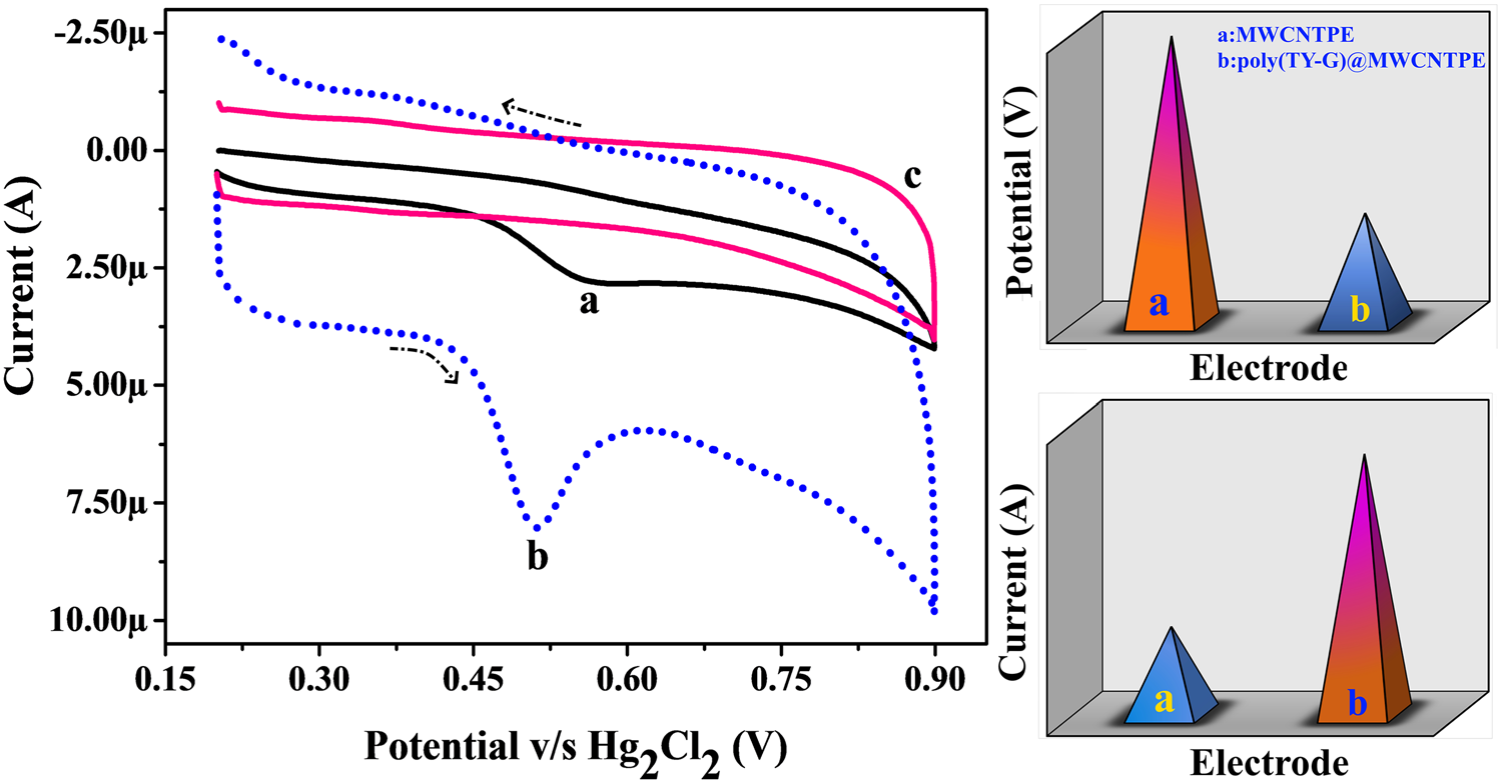
CVs for the presence and absence (Line ‘c’) of 0.005 mM FLFT in PB (0.2 M and 6.0 pH) on the surface of Poly(TY-G)LMWCNTPE (Line ‘b’) and MWCNTPE (Line ‘a’) at the potential scan rate of 0.1 V/s and the potential width of 0.2 to 0.9 V versus Hg_2_Cl_2_.

**Scheme 3:**
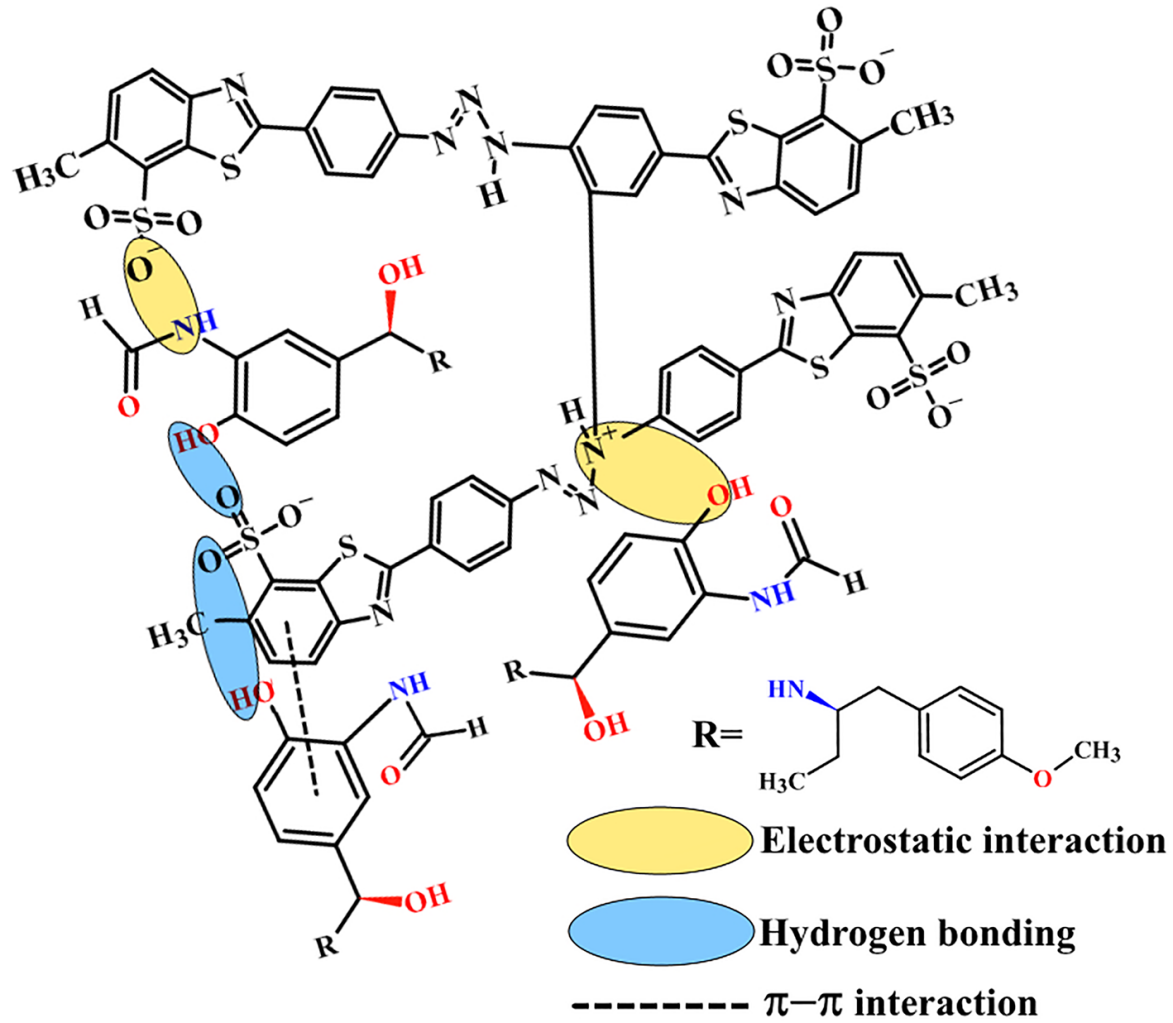
The probable scheme of π–π, hydrogen bonding, electrostatic, and dipole–dipole interactions between FLFT molecules and poly(TY-G).

The electro-oxidation nature of FLFT on the surface of bare MWCNTPE and Poly(TY-G)LMWCNTPE has been discussed by documenting the CVs at the optimum experimental incidents. Figure 8 parades the CVs at the potential scan rate of 0.1 V/s and the potential width of 0.2 to 0.9 V during the presence and absence (Line ‘c’) of 0.005 mM FLFT in PB (0.2 M and 6.5 pH) on the surface of Poly(TY-G)LMWCNTPE (Line ‘b’) and MWCNTPE (Line ‘a’). In the presence of FLFT, a fine electro-oxidation peak is viewed at the potential of 0.514 V as compared to bare MWCNTPE, which shows a less enhanced electro-oxidation peak at the potential of 0.569 V. Furthermore, modified electrode provides elevated electro-oxidation peak current and lesser electro-oxidation peak potential for FLFT than the bare MWCNTPE delivers an exceptional electro-catalytic potential. This shift in both the current and potential at the modified electrode is most conceivably due to the various interactions between FLFT molecules and poly(TY-G), such as π–π covalent, hydrogen bonding, electrostatic, and dipole–dipole interactions, shown in Scheme 3^37^. Furthermore, in the absence of FLFT in PB (0.2 M and 6.5 pH), the surface of Poly(TY-G)LMWCNTPE didn’t show any electro-catalytic oxidation response.

### Calibration plot for FLFT with and without UA

**Figure 9:**
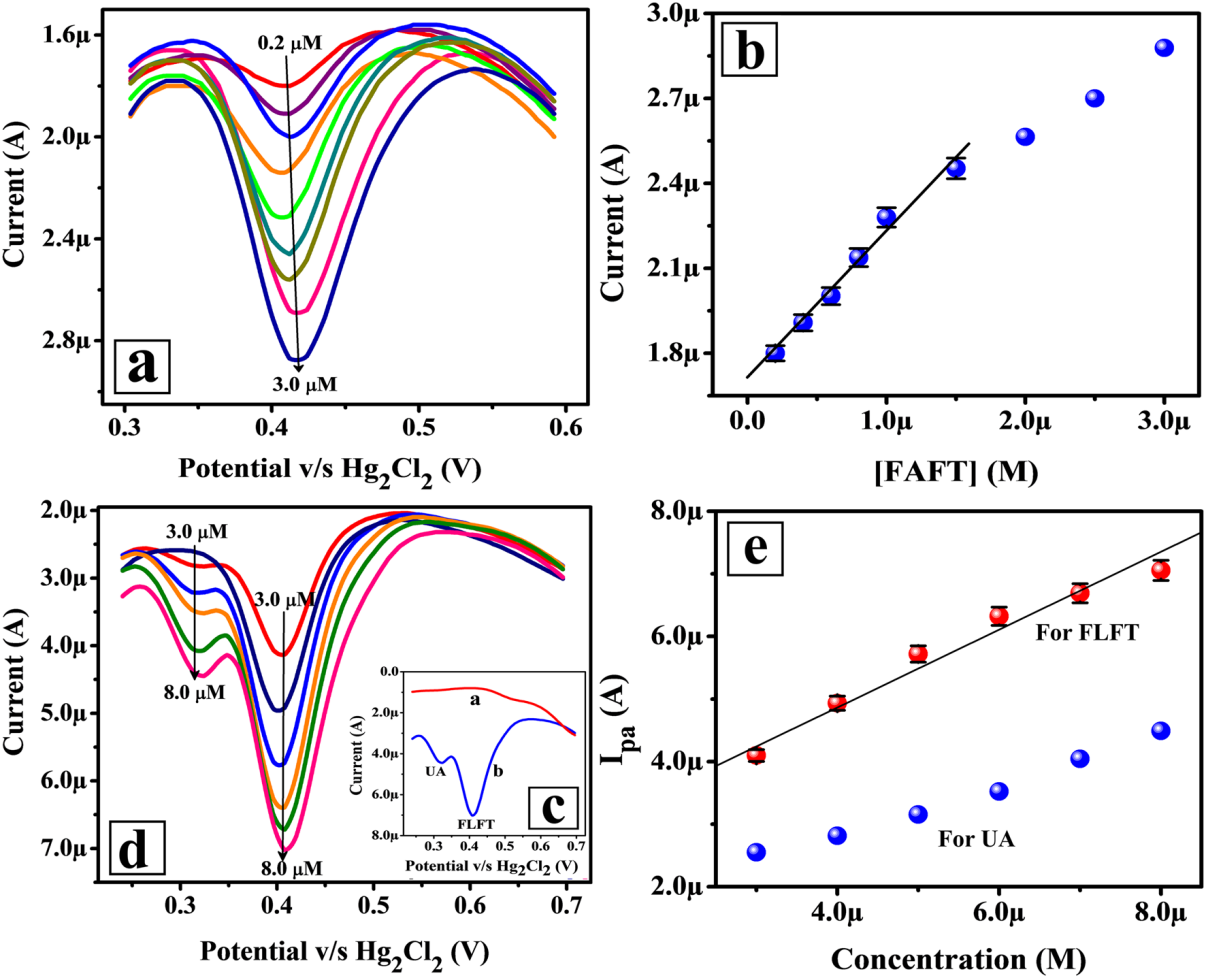
(**a**) DPVs for the different concentrations of FLFT in the range from 0.2 to 3.0 μM in PB (0.2 M and 6.5 pH) on the surface of Poly(TY-G)LMWCNTPE at the potential gap of 0.3 to 0.6 V versus Hg_2_Cl_2_. (**b**) The plot of the concentration of FLFT versus the electro-oxidation peak current of FLFT (n = 6). (**c**) DPVs for FLFT and UA in PB (0.2 M and pH 6.5) on the surface of Poly(TY-G)LMWCNTPE (Line ‘b’) and MWCNTPE (Line ‘a’) at the potential gap of 0.25 to 0.7 V versus Hg_2_Cl_2_. (**d**) DPVs for the different concentrations of FLFT and UA in the range from 0.3 to 0.8 μM in PB (0.2 M and pH 6.5) on the surface of Poly(TY-G)LMWCNTPE at the potential gap of 0.25 to 0.7 V versus Hg_2_Cl_2_. (**e**) The plot of the concentration of FLFT and UA versus the electro-oxidation peak current of FLFT and UA (n = 6).

The detection capability of the prepared Poly(TY-G)LMWCNTPE towards FLFT was verified based on the concentration variation method in the presence and absence of UA using the DPV technique (Inset Fig. 9). Figure 9a shows the documented differential pulse voltammograms (DPVs) of different concentrations of FLFT in the range from 0.2 to 3.0 μM in PB (0.2 M and pH 6.5) at the surface of Poly(TY-G)LMWCNTPE. Here, the electro-oxidation peak current of FLFT enhanced as the concentration of FLFT increased (Fig. 9b); also, the preliminary concentration range 0.2 to 1.5 μM gives a finer linear correlation and the achieved results are fitted in the following linear relation is as follows,

10 $${\text{I}}_{{{\text{pa}}}} \left( {\text{A}} \right) = 1.707 \times 10^{{ - 6}} + \left( {0.520 \pm 0.032} \right)\left[ {{\text{FLFT}}} \right]\left( {\text{M}} \right)\,\left({{\text{R}}^{2} = 0.999} \right).$$

The LOD and limit of quantification (LOQ) values are determined based on the slope of Eq. (10) and the standard deviation of the five consecutive blank cycles. The deliberated LOD and LOQ values were found to be 0.0128 µM and 0.0427 µM, respectively. The high sensing proficiency of the presented electrode associate with previous FLFT sensors is deliberated in Table 1^11,12,14,26,27^, proposing that the Poly(TY-G)LMWCNTPE provides the lowest/very nearer LOD.

**Table 1:** Contrast of earlier reported techniques, electrodes, and LODs of FLFT with the current work.

Method	Electrode	Buffer solution	Linear range (µM)	LOD (µM)	References
HPLC	–	Ammonium acetate with Acetic acid	1.0–50.0	0.071	^11^
IPC	–	Acetate	0.05–6.0	0.021	^12^
RP-HPLC	–	Acetonitrile	0.03–3.1	0.037	^14^
DPV	Suberic acid functionalized CuO NFs@glassy carbon electrode	Britton-Robinson	0.1–5.0	0.010	^26^
SWV^a^	Glassy carbon electrode	H_2_SO_4_	8.0–60.0	0.404	^27^
DPV				0.354	
DPV	Poly(TY-G)MMWCNTPE	PB	0.2–1.5	0.0128	Current effort

^a^Square wave voltammetry.

The simultaneous examination of FLFT and UA in PB (0.2 M and pH 6.5) was performed using the DPV technique on the surface of Poly(TY-G)LMWCNTPE (Line ‘b’) and MWCNTPE (Line ‘a’) (Fig. 9c). The line ‘b’ discloses well and distinctive electro-oxidation peaks resultant to UA and FLFT at the electro-oxidation peak potentials of 0.321 V and 0.409 V, individually with the lack of a background current. Nevertheless, at MWCNTPE the individual peak split-up is not definite for FLFT and UA. This consequence states that Poly(TY-G)LMWCNTPE is a sensitive tool for the selective and innovative assessment of FLFT in the occurrence of UA. Figure 9d displays the recorded DPVs of different concentration of FLFT and UA in the range from 3.0 to 8.0 μM in PB (0.2 M and pH 6.5) at Poly(TY-G)LMWCNTPE. The electro-oxidation peak current of FLFT and UA get promoted as the concentration of FLFT and UA increased (Fig. 9e) and FLFT offers a better linear association in the presence of UA and the results are fitted in the following relation is as follows,

11 $${\text{I}}_{{{\text{pa}}}} \left( {\text{A}} \right) = 2.375 \times 10^{{ - 6}} + \left( {0.622 \pm 0.030} \right)\left[ {{\text{FLFT}}} \right]\left( {\text{M}} \right)\left( {{\text{R}}^{2} = 0.989} \right).$$

The determined LOD value was found to be 0.0129 µM, suggesting that the Poly(TY-G)LMWCNTPE delivers almost nearer LOD value even in the presence of interfering UA molecule. Hence, the prepared electrode is almost free from interference.

### Compatibility of the Poly(TY-G)LMWCNTPE

**Figure 10:**
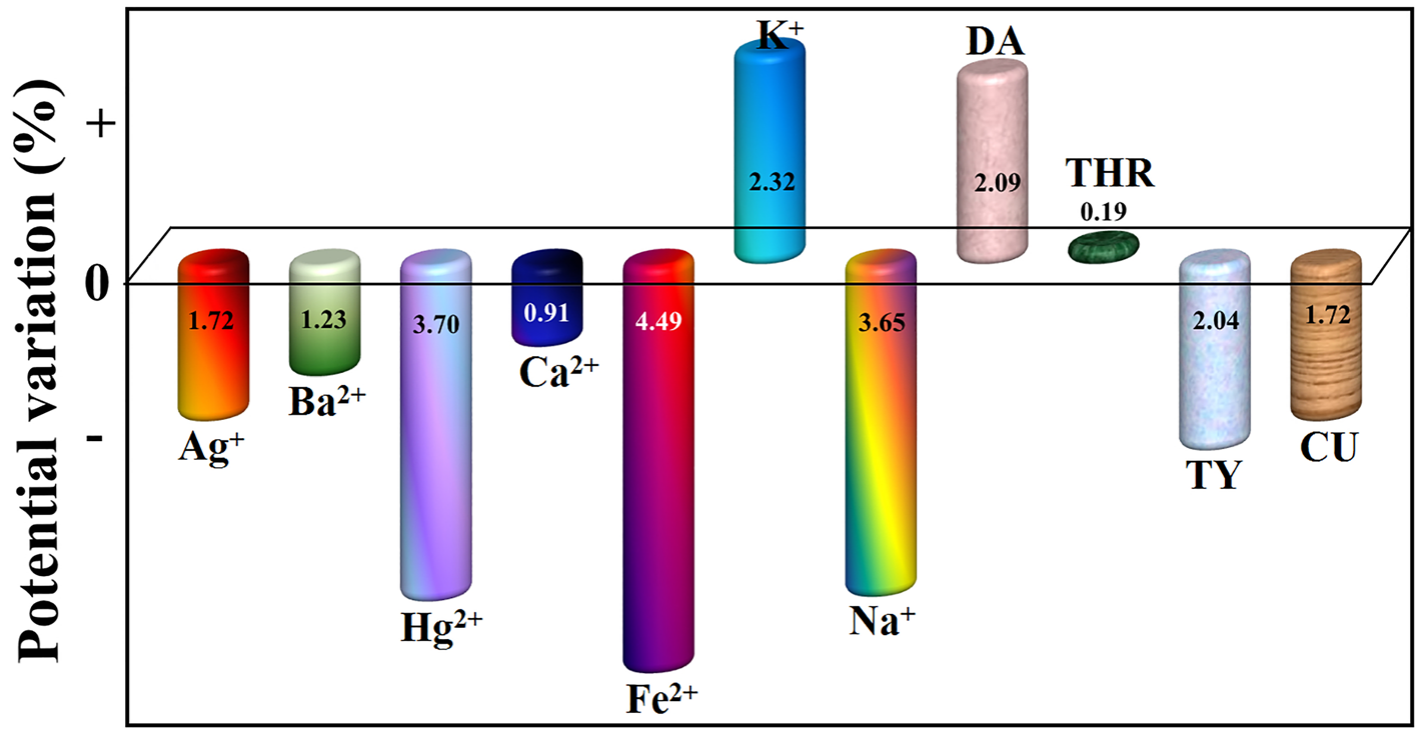
The plot of the different chemical species (Ag^+^, Ba^2+^, Hg^2+^, Ca^2+^, Fe^2+^, K^+^, Na^+^, DA, THR, TY, and CU) versus the electro-oxidation potential variation of FLFT in PB (0.2 M and 6.5 pH).

The compatibility of the Poly(TY-G)LMWCNTPE towards the electro-oxidation of FLFT was examined via the DPV method in the presence of some biologically available species (metal ions and organic molecules, each having a concentration of 1.0 mM). Figure 10 shows the graph of different chemical species (such as Ag^+^, Ba^2+^, Hg^2+^, Ca^2+^, Fe^2+^, K^+^, Na^+^, dopamine (DA), tryptophan (THR), tyrosine (TY), and curcumin (CU)) versus the % of electro-oxidation potential variation of FLFT. Here we observe only ± 5.0% of potential variation at the oxidation of FLFT, suggesting that Poly(TY-G)LMWCNTPE shows acceptable compatibility in presence of the above-mentioned chemical species. But in the case of electro-oxidation peak current, the compatibility was little vary compared to the base peak current.

### Analysis of FLFT in medicinal sample

In supplement to the aforesaid studies, the Poly(TY-G)LMWCNTPE sensing capability towards FLFT was determined in a medicinal sample based on the standard addition approach. As from Table 2, the five consecutive FLFT concentrations in the range from 0.6 to 2.0 µM give a fine recovery in the range from 90.64 to 96.70% with a good LOD (0.150 µM) and relative standard deviation (RSD) (1.87%). These obtained data show the good sensing ability of Poly(TY-G)LMWCNTPE for FLFT analysis in a medicinal sample.

**Table 2:** Result of percentage recovery, LOD, and RSD of FLFT at Poly(TY-G)LMWCNTPE in a medicinal sample.

Sample	Added (µM)	Found (µM)	Recovery (%)	LOD (µM)	RSD (%)
FLFT capsule (n = 5)	0.60	0.58	96.37	0.150	1.876
	0.80	0.77	96.70		
	1.00	0.91	91.22		
	1.50	1.35	90.64		
	2.00	1.87	93.85		

### Repeatability, reproducibility, and stability of Poly(TY-G)LMWCNTPE

Repeatability, reproducibility, and stability are the supplemental factors to reveal the consistency and practicability of a used method and developed electrode. The repeatability of Poly(TY-G)LMWCNTPE was analyzed by cycling five sequential CV cycles for 0.005 mM FLFT in 0.2 M PB of pH 6.5 (renewed at the end of each cycle) on the surface of constantly fixed Poly(TY-G)LMWCNTPE. The found RSD value of 1.36% delivers a fine repeatability of the used method and the developed electrode towards the FLFT oxidation. The reproducibility of Poly(TY-G)LMWCNTPE was studied by cycling continual five CV cycles for constantly fixed 0.005 mM FLFT in PB (0.2 M and pH 6.5) on the surface of Poly(TY-G)LMWCNTPE (renewed at the end of each CV cycle). The found RSD value of 2.16% offers a good reproducibility for the operated method and developed electrode towards the oxidation of FLFT. Likewise, the storage stability of the developed electrode was tested towards 0.005 mM FLFT in PB (0.2 M and pH 6.5) via the DPV method by quantifying the current response at the Poly(TY-G)LMWCNTPE (initial current and current after one day). The calculated value of the percentage of degradation gives 90.72% of retained peak current affords a first-class electrode storage stability.

## Conclusions

In this effort, the Poly(TY-G)LMWCNTPE and MWCNTPE were prepared simplistically and economically for the detection of FLFT in PB using the CV and DPV methodologies. The modification of poly(TY-G) on MWCNTPE was confirmed by FE-SEM, EIS, CV, electro-active surface area, and surface concentration measurements. The enhanced active surface area of the Poly(TY-G)LMWCNTPE improves the rate of electron and proton transfer during the electro-oxidation of FLFT. The CV outcomes validate different interactions between FLFT molecules and Poly(TY-G)LMWCNTPE, which significantly shifts the peak current to a higher level and peak potential to a lower level in the oxidation of FLFT. The impact of the potential scan rate study reveals the heterogeneous rate constant and diffusion kinetics (also supported by the effect of accumulation) with the transfer of 2 e^−^ and 2 H^+^ in the electro-oxidation of FLFT. The supporting electrolyte pH study shows that the proportion of electron and proton transfer in the electro-oxidation of FLFT is equivalent (1:1). The Poly(TY-G)LMWCNTPE implicates better electrochemical behavior (in presence and absence of UA) with fine linear response, superior sensitivity with lower LOD, first-class selectivity (in the presence of different chemical species), reproducibility, repeatability, and good storage stability. Additionally, the modified Poly(TY-G)LMWCNTPE provides excellent recovery (90.64 to 96.70%) for FLFT in a medicinal sample. All these results concluded that the Poly(TY-G)LMWCNTPE is a more optimistic and potent sensing tool in the analysis of many other electro-active molecules and medicinal samples in vision of its superior active surface area and electrocatalytic activity.
